# Nebulised Isotonic Hydroxychloroquine Aerosols for Potential Treatment of COVID-19

**DOI:** 10.3390/pharmaceutics13081260

**Published:** 2021-08-14

**Authors:** Waiting Tai, Michael Yee Tak Chow, Rachel Yoon Kyung Chang, Patricia Tang, Igor Gonda, Robert B. MacArthur, Hak-Kim Chan, Philip Chi Lip Kwok

**Affiliations:** 1 Advanced Drug Delivery Group, Sydney Pharmacy School, Faculty of Medicine and Health, The University of Sydney, Camperdown, NSW 2006, Australia; wtai6746@uni.sydney.edu.au (W.T.); yee.chow@sydney.edu.au (M.Y.T.C.); yoon.chang@sydney.edu.au (R.Y.K.C.); patricia.tang@sydney.edu.au (P.T.); kim.chan@sydney.edu.au (H.-K.C.); 2 Pulmoquine Therapeutics, Inc., 1155 Camino Del Mar Suite 481, Del Mar, CA 92014, USA; igonda@pulmoquine.com (I.G.); rmacarthur@rockefeller.edu (R.B.M.)

**Keywords:** hydroxychloroquine, coronavirus disease 2019 (COVID-19), vibrating mesh nebuliser, inhalation, aerosol, droplet

## Abstract

The coronavirus disease 2019 (COVID-19) is an unprecedented pandemic that has severely impacted global public health and the economy. Hydroxychloroquine administered orally to COVID-19 patients was ineffective, but its antiviral and anti-inflammatory actions were observed in vitro. The lack of efficacy in vivo could be due to the inefficiency of the oral route in attaining high drug concentration in the lungs. Delivering hydroxychloroquine by inhalation may be a promising alternative for direct targeting with minimal systemic exposure. This paper reports on the characterisation of isotonic, pH-neutral hydroxychloroquine sulphate (HCQS) solutions for nebulisation for COVID-19. They can be prepared, sterilised, and nebulised for testing as an investigational new drug for treating this infection. The 20, 50, and 100 mg/mL HCQS solutions were stable for at least 15 days without refrigeration when stored in darkness. They were atomised from Aerogen Solo Ultra vibrating mesh nebulisers (1 mL of each of the three concentrations and, in addition, 1.5 mL of 100 mg/mL) to form droplets having a median volumetric diameter of 4.3–5.2 µm, with about 50–60% of the aerosol by volume < 5 µm. The aerosol droplet size decreased (from 4.95 to 4.34 µm) with increasing drug concentration (from 20 to 100 mg/mL). As the drug concentration and liquid volume increased, the nebulisation duration increased from 3 to 11 min. The emitted doses ranged from 9.1 to 75.9 mg, depending on the concentration and volume nebulised. The HCQS solutions appear suitable for preclinical and clinical studies for potential COVID-19 treatment.

## 1. Introduction

Since December 2019, the world has been adversely affected by the coronavirus disease 2019 (COVID-19) pandemic, caused by severe acute respiratory syndrome coronavirus 2 (SARS-CoV-2). As of 17 July 2021, there were 189,482,312 confirmed cases globally, with 4,074,668 deaths [1]. These tolls continue to increase daily at alarming rates. In addition to stressing public health systems, the pandemic has wreaked havoc on the economy and people’s livelihoods worldwide. Although various vaccines have been developed and inoculation programmes are progressively being launched in different countries, their effectiveness in achieving general population immunity and reducing viral transmission needs to be yet evaluated [2,3,4]. Vaccines may not completely restore the present situation to the pre-COVID-19 “norm” [4]. In addition, the long-term safety of the vaccines is yet unclear due to the accelerated development of these products [5]. Public perception of the potential harm from the vaccines versus that from the infection will inevitably influence vaccination rate [6]. Furthermore, various vaccines have been reported to be less effective against SARS-CoV-2 variants, which has prompted questions on how the efficacy of future vaccines can be maintained to tackle incessant viral mutations [7,8,9,10,11]. Therefore, although vaccines are essential, their use alone may not be sufficient to solve the crisis. Drugs for treating the infection are required as a pragmatic strategy. The more effective drugs that are available, the better the health sector is equipped to combat this pandemic.

The course of COVID-19 progresses through two clinical phases. The early phase is predominated by viral replication, whereas the late phase features uncontrolled inflammatory or immune responses to the virus, leading to tissue damage [12]. Thus, the mode of treatment for COVID-19 depends on the stage of the disease, with antiviral and anti-inflammatory therapies being more effective in the early and late phases, respectively. The United States National Institutes of Health advises using anti-SARS-CoV-2 monoclonal antibodies (casirivimab + imdevimab combination or sotrovimab alone) for non-hospitalised patients with mild to moderate COVID-19 at high risk of disease progression [12]. Remdesivir is hitherto the only antiviral drug approved for treating COVID-19 by the United States Food and Drug Administration and European Medicines Agency [13,14]. It is recommended for hospitalised patients on supplemental oxygen and can be used with dexamethasone if oxygen requirement is moderately high [12]. For recently hospitalised patients requiring systemic inflammation using high-flow oxygen or non-invasive ventilation, baricitinib or tocilizumab can be added to dexamethasone with or without remdesivir. Dexamethasone is used alone in the most serious cases, when the patient requires invasive mechanical ventilation or extracorporeal membrane oxygenation [12]. In addition to the drugs mentioned above, the United States Food and Drug Administration has also issued emergency use authorisation for the bamlanivimab + etesevimab combination for mild to moderate COVID-19 [14]. The American Society of Health-System Pharmacists has issued a comprehensive list of approved and experimental drugs for COVID-19 with their clinical evidence that is constantly updated [15]. Some of those drugs (e.g., hydroxychloroquine, azithromycin, lopinavir, ritonavir) have been or are being investigated for repurposing for COVID-19 [16,17,18,19]. In particular, hydroxychloroquine is an old 4-aminoquinoline antimalarial chemically similar to, but less toxic than, chloroquine [17,20]. It has been employed for decades for treating autoimmune conditions such as rheumatoid arthritis and lupus erythematosus, due to its immunomodulatory effects [21]. It is administered as hydroxychloroquine sulphate (HCQS) because this salt form is freely soluble in water (aqueous solubility of 1 in 5), with 1 mg of HCQS being equivalent to about 0.775 mg of the base [21,22]. Absorption from the gastrointestinal tract is rapid and extensive [23]. Then, it undergoes hepatic first pass metabolism and results in an oral bioavailability of 79% [24,25].

The proposed use of hydroxychloroquine to prevent and treat COVID-19 is based on its antiviral and immunomodulatory effects reported in the literature. Hydroxychloroquine and chloroquine showed in vitro antiviral activity against SARS-CoV-2 before and after infection in Vero cells, which were derived from the kidney epithelial cells isolated from an African green monkey [26,27]. When the cells were pre-treated with the drugs for 2 h before infection, the half maximal effective concentration (EC_50_) of hydroxychloroquine and chloroquine for inhibiting viral replication after 48 h of incubation was 5.85 and 18.01 µM, respectively [27]. On the other hand, their EC_50_ was 0.72 and 5.47 µM, respectively, when they were added after infecting with the virus at a multiplicity of infection (MOI) of 0.01. In another study, their EC_50_ on Vero E6 cells (ATCC-1586) at the same MOI was 4.51 and 2.71 µM, respectively [28]. The different EC_50_ for both drugs between the two studies might be due to the different Vero cell lineages used. Nevertheless, those levels were not lethal to the cells because they were significantly lower than the half-maximal cytotoxic concentrations (CC_50_) on Vero E6 cells (249.50 and 273.20 µM for hydroxychloroquine and chloroquine, respectively) [28]. From these in vitro data, hydroxychloroquine and chloroquine may potentially be used for the prophylaxis and treatment of COVID-19.

Although the antiviral mechanism of these drugs is unclear, they have been shown to prevent the attachment of SARS-CoV-2 to angiotensin-converting enzyme 2 (ACE-2), sialic acid-containing glycoproteins, and gangliosides on the surface of host cells to which the virus needs to bind for entry [29,30]. In addition, the drugs are weak bases so they increase the pH of the normally acidic endosomes and lysosomes in host cells [17,20,29,30]. The alkalinisation alters the homoeostasis of these intracellular organelles and hinders various processes in the viral life cycle that depend on them (e.g., cell entry, replication, release) [29,30].

The initial local airway inflammation in COVID-19 may lead to hypercytokinaemia, or “cytokine storm”, the uncontrolled upregulation of multiple pro-inflammatory cytokines such as interleukin (IL)-1β, IL-6, IL-8, tumour necrosis factor-α, and granulocyte-colony stimulating factor [31,32]. Then, the resultant hyperinflammation may cause pulmonary fibrosis, hypoxaemia, damage to other organs, and death. Hydroxychloroquine and chloroquine have long been known to inhibit the production of some of the pro-inflammatory cytokines [33,34]. Therefore, their use in COVID-19 may be beneficial, especially when administered early in the disease to prevent the induction of a cytokine storm and further deterioration of health [25,27,35]. In fact, early treatment of COVID-19 patients with orally administered hydroxychloroquine within one day of hospitalisation (400 mg twice a day on Day 1, followed by 200 mg twice a day on Days 2 to 5) decreased their risk of being transferred to intensive care units by 53%, which is attributed to the anti-inflammatory properties of the drug [36].

Despite the points discussed above suggesting the potential usefulness of hydroxychloroquine in COVID-19, in vivo evidence supporting its clinical application is still lacking. Its benefits were not observed in cynomolgus macaques or human patients infected with SARS-CoV-2 [2,17,18,19,24,30,37]. This might be due to shortcomings in the route of administration and trial design employed in the clinical studies. The drug was administered orally in all the cases because it is conventionally formulated as tablets. Hydroxychloroquine has a large volume of distribution (5522 L and 44,257 L calculated from blood and plasma data from healthy adults, respectively) and a long terminal elimination half-life of about 40 days as it extensively distributes into and remains in body tissues [38]. Delivering this drug via the oral route is inefficient when the lungs are the primary delivery target site. To achieve a therapeutic drug concentration in the airways, the oral dose needs to be sufficiently high to compensate for the drug loss due to first pass metabolism and distribution into other organs. However, high doses will increase the risk of systemic adverse effects. Indeed, oral hydroxychloroquine has been reported to cause cardiac toxicity including QT prolongation and ventricular arrhythmias in both COVID-19 patients and patients with other illnesses (rheumatoid arthritis, lupus erythematosus, malaria) [21,24,30]. This risk may be further heightened when co-administered with azithromycin [18,24,30]. The lack of robustness of the clinical studies further complicates data interpretation. A number of them were not properly controlled, randomised, or blinded [30]. The patient sample size of some trials was too small for statistical power, while the disease severity amongst certain subject groups varied widely, so it was difficult to interpret the results. Some published studies were not peer-reviewed, especially those from early 2020 as urgent dissemination of medical information on COVID-19 was prioritised [30]. The emergency nature of the pandemic may have imposed limitations on the design and execution of the studies, consequently impacting data quality. Due to its anti-inflammatory properties, inhalation delivery of hydroxychloroquine solutions was tested in sheep for the treatment of asthma [39]. Later research with a soft mist inhaler using an aqueous formulation of HCQS proceeded into humans [40]. The antiviral and anti-inflammatory activities of this drug against rhinoviral infection in human bronchial cells were reported [41].

Robust, controlled clinical trials for hydroxychloroquine utilising a more efficient route of administration are required to better evaluate its efficacy in COVID-19. Since the respiratory tract is the initial site of infection and inflammation, direct inhalation delivery is better targeted than oral administration, as lower doses can be used to achieve high local drug concentrations in the airways to maximise therapeutic action and minimise systemic adverse effects [25,42,43,44]. Based on the in vitro extracellular concentrations in the activity assays against SARS-CoV-2, direct delivery by inhalation is necessary to achieve adequate HCQS concentration in the upper and central airway target tissues to be effective [45]. Our research group recently characterised jet milled, crystalline HCQS powders deliverable from dry powder inhalers for clinical testing [43]. However, nebulised solutions offer more flexible dose adjustment. Since inhaled dry powders in general and particularly those of HCQS are known to cause coughing [46,47], the use of an isosmotic, pH-neutral formulation was preferable. Nebulisers can be used by patients of all ages, including those who are ventilated. Furthermore, the safety of nebulised HCQS solutions was previously demonstrated in healthy volunteers as well as subjects with pulmonary disease [40,48]. Thus, this paper reports on isotonic HCQS solutions that can be prepared, sterilised, and nebulised for potential treatment of COVID-19.

## 2. Materials and Methods

### 2.1. Chemicals

HCQS powder of United States Pharmacopoeia (USP) grade (Lot 1910P031, Batch 033600-192021) was purchased from Sci Pharmtech Inc. (Taoyuan, Taiwan). Chromatographic grade methanol and acetonitrile were bought from RCI Labscan (Bangkok, Thailand) and Honeywell (Morris Plains, NJ, USA), respectively. Deionised water was obtained from a MODULAB^®^ High Flow Water Purification System (Evoqua Water Technologies, Pittsburgh, PA, USA).

### 2.2. HCQS Nebulised Solutions

Isotonic and pH-neutral solutions containing 20, 50, and 100 mg/mL of HCQS were prepared in volumetric flasks. Then, they were transferred to 50 mL of polypropylene centrifuge tubes (Corning, Corning, NY, USA) and stored in darkness at ambient temperature until use. The osmolality of the solutions was measured with a K-7000 vapor pressure osmometer (Knauer, Berlin, Germany). The cell and head temperatures were set as 60 °C and 62 °C, respectively, and allowed to stabilise for an hour before use. These temperatures followed those recommended in the instrument manual for calibrating and measuring sodium chloride aqueous solutions [49]. The measurement time and gain were 1.5 min and 16, respectively. Approximately 1 mL of each sample solution was drawn into glass microsyringes and inserted into the osmometer. One droplet from each sample was dispensed onto the thermistor for each osmolality measurement. The droplet was replaced by a new one when repeating the measurement. The experiments were conducted in quadruplicate (*n* = 4) for each HCQS solution. The target osmolality range was 260–360 mOsmol/kg H_2_O [50,51]. The pH of the solutions was measured with a pH 700 benchtop meter (Oakton, Vernon Hills, IL, USA). The target pH range was 6.8–7.5 [50,51].

### 2.3. High Performance Liquid Chromatography (HPLC)

HCQS was quantified by a modified reverse phase-HPLC method from the USP [52]. The assay was performed on an automated HPLC system that consisted of a DGU-20A degassing unit, a LC-20AT HPLC pump, a SIL-20A HT autosampler, a CTO-20A column oven, and an SPD-20A UV detector (Shimadzu, Kyoto, Japan). The mobile phase was composed of 10:10:80:0.2 by volume of methanol, acetonitrile, 0.12 g/L sodium 1-pentanesulfonate monohydrate aqueous solution, and orthophosphoric acid. The mobile phase and all other solvents were filtered and degassed before use. The Agilent Zorbax SB-C18 column (5 µm, 4.6 × 250 mm; Agilent, Santa Clara, CA, USA) was kept at 35 °C during the runs. Each sample ran for 15 min at a mobile phase flow rate of 1 mL/min. The injection volume and detection wavelength were 20 μL and 254 nm, respectively. Standard solutions (6.25–1000 µg/mL) were freshly prepared by serially diluting a 100 mg/mL HCQS solution aliquot that had been filtered through a sterile Millex-GP 0.22 µm hydrophilic polyethersulfone membrane syringe filter (Millipore, Burlington, MA, USA) (see below for the method). The diluent for the standard solutions was deionised water and 50:50 *v*/*v* methanol/water, depending on the diluent used for the samples.

### 2.4. Effect of Filtration on HCQS Solutions

The effect of filtration on the drug concentration, osmolality, and pH was investigated because the HCQS solutions would be sterilised by filtration before nebulisation. Approximately 3 mL of the 20, 50, or 100 mg/mL HCQS solution was drawn into a 3 mL syringe (Terumo, Tokyo, Japan). Then, a sterile Millex-GP 0.22 µm hydrophilic polyethersulfone membrane syringe filter was attached to the syringe. About 1 mL of the solution was ejected through the filter and discarded. The remaining 2 mL in the syringe was filtered and collected into a 2 mL microcentrifuge tube (Quality Scientific Plastics, Petaluma, CA, USA). The drug concentration, osmolality, and pH of the unfiltered and filtered solutions were measured as outlined above. For the HPLC runs, all samples were diluted with deionised water to 500 μg/mL to be within the concentration range of the standard curve.

### 2.5. Recovery of HCQS from SureGard Filters

SureGard filters (Bird Healthcare, Bayswater, VIC, Australia) were used in the dose output and cascade impaction runs (connected between the impactor and the vacuum pump) to collect the nebulised droplets so the recovery of HCQS from this type of filter was investigated. These filters were spiked with 2 or 75 mg of HCQS by adding 20 or 750 µL of a 100 mg/mL HCQS nebulised solution to a new filter, respectively. The openings of the filter were sealed with Parafilm (Bemis, Oshkosh, WI, USA) after adding 10 mL of deionised water or 50:50 *v*/*v* methanol/water. The filters were immediately shook by hand for 5 min or left to stand for 30 min first, followed by 5 min of shaking. The samples were diluted 10-fold with deionised water or 50:50 *v*/*v* methanol/water accordingly before HPLC assay.

### 2.6. Dose Output

**Figure 1:**
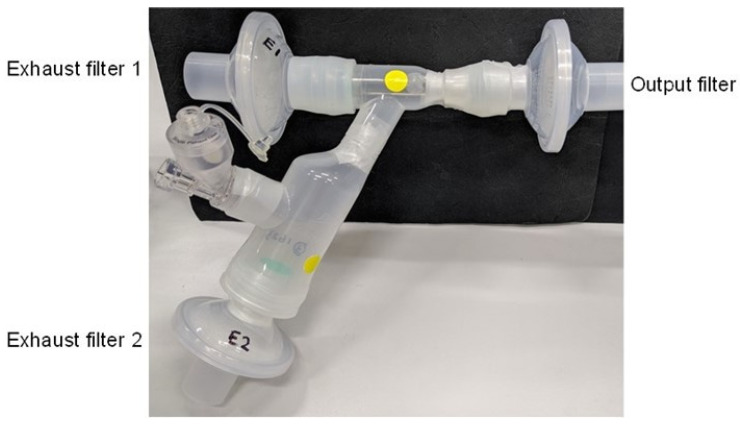
Setup for the dose output runs.

The HCQS dose output from three new Aerogen^®^ Solo nebulisers (Mesh numbers C1901059-0822, C1901059-0797, and C1901059-1623; Aerogen, Galway, Ireland) was measured with individualised Aerogen Ultra aerosol chambers. These nebulisers plus aerosol chambers will be referred to in this report as Nebulisers 1, 2, and 3, respectively. The same Aerogen controller was used for all experiments. One SureGard filter was connected to the outlet of the Aerogen Ultra mouthpiece. A filter was also fitted to the exhaust end of the mouthpiece and the exhaust port at the bottom of the Aerogen Ultra (Figure 1). Thus, there were one outlet filter and two exhaust filters. Silicone adaptors were used to connect the mouthpiece to the outlet and one of the exhaust filters (Figure 1). The experiments were conducted under ambient conditions (18–25 °C, 20–65% RH).

The procedure followed the USP method [52] except that the aerosols were collected from the start to the end of nebulisation instead of collecting them for the first minute using one output filter and then collecting the rest of the aerosols with another output filter. This was to avoid drug loss when changing the filters. It would also simplify the experimental procedure. The output filter was not overloaded by the lengthening of collection duration. The end of nebulisation was determined by visual inspection when no solution remained in the nebuliser.

Nebulised dose output was measured for the following HCQS solutions. The same scheme was adopted for the laser diffraction and cascade impaction experiments (see below).

- 1 mL of 20 mg/mL;
- 1 mL of 50 mg/mL;
- 1 mL of 100 mg/mL;
- 1.5 mL of 100 mg/mL.

HCQS solution was added into the reservoir of the Aerogen Solo by pipetting. The PWG-33 breathing simulator (Piston Medical, Budapest, Hungary) was connected to the output filter. The simulated breathing waveform was sinusoidal at 15 cycles/minute, with an inhalation-to-exhalation ratio of 1:1 and a tidal volume of 500 mL [52]. The outlet and exhaust filters captured droplets exiting the nebulisers during the inhalation and exhalation phases in the breathing cycle, respectively. The nebuliser and breathing simulator were operated from the start to the end of nebulisation, after which the setup was left to stand for 20 min before being removed and assayed. This was to allow the droplets in the Aerogen Ultra to settle by gravitational sedimentation and avoid potential aerosol loss if the setup was disassembled immediately. The runs were conducted in triplicate for each nebuliser.

The openings of the two exhaust filters were sealed with Parafilm after adding in 10 mL of deionised water. Then, the exhaust filters were exhaustively rinsed by shaking for 5 min. The outlet filter was placed into a 600 mL glass beaker. Four hundred millilitres of deionised water, a glass weight, and magnetic stirrer were added into that beaker afterwards. The glass weight was to weigh down the filter to ensure its complete immersion in the water. The mixture was magnetically stirred for 5 min, followed by shaking for another 5 min. The liquid reservoir and outlet of the Aerogen Solo were exhaustively washed with 10 mL of deionised water and 6 min of shaking in total. The same was performed on the two silicone adaptors. The washings were collected into a 100 mL volumetric flask. The openings of the Aerogen Ultra were sealed with Parafilm after adding in about 10 mL of deionised water. The whole chamber was exhaustively rinsed in the same manner as described for Aerogen Solo. All washings were pooled into the same volumetric flask. The volume was made up to 100 mL with deionised water. All samples were assayed by HPLC.

### 2.7. Laser Diffraction

The nebulised droplets were sized by laser diffraction using Spraytec (Malvern Panalytical, Malvern, UK) with an inhalation cell and at an acquisition frequency of 2.5 kHz. The outlet of the Aerogen Ultra mouthpiece was positioned 1 cm from the laser measurement zone to minimise evaporation during measurement. A vacuum pump connected to the other end of the inhalation cell with entrained dilution air was used to remove the aerosols continuously to (1) prevent droplet re-entrainment of droplets into the laser measurement zone; and (2) maintain the laser signal transmission >70% to minimise multiple scattering. The Aerogen Ultra mouthpiece was not sealed to the inhalation cell, so the airflow through the Aerogen Ultra was unknown. Signals from Detectors 1–10 were excluded to account for beam steering effects. The real and imaginary refractive indices for the droplets were taken to be the same as those for water, which were 1.33 and 0.00, respectively. The refractive index for air was 1.00. These values were deemed appropriate because all measurements showed low residual values (<0.5%). The droplets were sized when the signal transmission was <99%. The duration of nebulisation was the time that aerosols were seen by eye to traverse continuously through the laser measurement zone. The raw data of each run were processed to yield an averaged volumetric diameter distribution, from which the volumetric median diameter (VMD) and geometric standard deviation (GSD) were derived. The percentage of aerosol sample by volume under 1, 2, 3, 5, and 10 µm were also calculated.

### 2.8. Cascade Impaction

The aerosol performance of the three Aerogen Solo nebulisers coupled to their respective Aerogen Ultra aerosol chambers was measured by the USP method using a Next Generation Impactor (NGI; USP Apparatus 5; Copley, Nottingham, UK) without a pre-separator [52]. The same Aerogen controller was used for all the experiments. The NGI and throat were chilled at 5 °C for at least 90 min beforehand. After chilling, a SureGard filter was connected to the NGI after the micro-orifice collector (MOC) to capture any drug that passed beyond the lowest impactor stage. The sealing of the apparatus was verified before each run by a vacuum leak test, after which the airflow rate was set to 15 L/min. A silicone adaptor was used to connect the mouthpiece to the USP induction port (throat). The experiments were conducted under ambient conditions (18–25 °C, 20–65% RH).

HCQS solution was added into the reservoir of the Aerogen Solo by pipetting. No exhaust filters were required to be connected to the Aerogen Ultra because the airflow was suction only. The nebuliser and vacuum pump were operated from the start to the end of nebulisation. The end of nebulisation was determined by visual inspection when no solution remained in the nebuliser. The setup was left to stand for 20 min before being removed and assayed. The co-solvent used for all NGI samples was 50:50 *v*/*v* methanol:water. For the 20 mg/mL HCQS runs, the Aerogen Solo, and Aerogen Ultra were exhaustively washed with this co-solvent, collected into a 100 mL volumetric flask, and made up to volume. The post-NGI filter was washed with 10 mL of the co-solvent, as for the dose output exhaust filter. The adaptor, throat, and NGI impactor stages were washed with 4 mL of the co-solvent. The assay for the 100 mg/mL HCQS runs was conducted in the same manner, except that Stages 1–6 were washed with 20 mL instead of 4 mL of the co-solvent.

The loaded dose was the amount of HCQS added into the nebuliser. The emitted dose was the total amount of drug assayed from the adaptor to the post-NGI filter. The recovered dose was the total amount of HCQS assayed on all the parts in the experimental setup, i.e., from the nebuliser to the post-NGI filter. Fine particle doses (FPDs) under 1, 2, 3, 5, and 10 µm were calculated, from which the corresponding fine particle fractions (FPFs) with respect to the loaded, emitted, and recovered doses were then derived. Likewise, the mass median aerodynamic diameter (MMAD) and GSD with respect to the recovered dose and the emitted dose were calculated. The MMAD was the diameter at 50% undersize interpolated from the cumulative recovered and emitted doses. The GSD was calculated by dividing the MMAD by the diameter at 16% undersize, which was in turn interpolated from the cumulative recovered and emitted doses.

### 2.9. Measurement of the Density of HCQS Solutions

The density of HCQS solutions (20, 50, and 100 mg/mL) was measured by first weighing deionised water in a 10 mL volumetric flask, filled to the mark. After discarding the water and drying the volumetric flask, HCQS solution was added to the mark and weighed. The density of the HCQS solutions was calculated with the following equation.

*ρ*_*H*_ = *ρ*_*W*_ (*m_H_*/*m_w_*) (1)

where *ρ_H_* and *ρ_W_* are the densities of HCQS solution and deionised water, respectively; and *m_H_* and *m_W_* are the masses of HCQS solution and deionised water in the 10 mL volumetric flasks, respectively. The density of deionised water at 24 °C, at which the measurements were conducted, was interpolated from the water density data in the CRC Handbook of Chemistry and Physics [53]. Three volumetric flasks were used to obtain triplicate measurements for each solution. The densities of the HCQS solutions were used to convert the volumetric diameters measured by laser diffraction to a volumetric aerodynamic diameter by the following equation [54].

*d_a_* = *d_v_*(*ρ_H_*/*ρ*_0_)^0.5^ (2)

where *d_a_* and *d_v_* are aerodynamic and volumetric diameters, respectively; and *ρ_0_* is unit density (1 g/cm^3^). The volumetric aerodynamic diameter was used for comparing the droplet sizes measured by laser diffraction to those by cascade impaction.

### 2.10. Statistical Analysis

One-way analysis of variance followed by Tukey’s post hoc test were performed using the SPSS software (IBM, Armonk, NY, USA). Statistical differences were indicated by *p* < 0.05 and α < 0.05.

## 3. Results

### 3.1. Effect of Filtration on Drug Concentration, Osmolality, and pH

Two batches of 100 mg/mL solutions were made (Batches A and B). Batch A was used to obtain the 20 mg/mL solution by dilution, while Batch B was used directly for the 100 mg/mL experiments and for making the 50 mg/mL solution. No drug degradation was observed over the 15 days during which all the experiments were performed. The drug concentration, osmolality, and pH of the HCQS solutions before and after filtration are presented in Table 1.

The osmolality and pH of all solutions were within the target ranges, regardless of filtration. The five-fold dilution of the Batch A 100 mg/mL solution to 20 mg/mL reduced the osmolality from 323.0 to 286.5 mOsmol/kg H_2_O, but it was still within the target range. HCQS concentration was not affected by filtration. On the other hand, osmolality and pH decreased after filtration, but the difference was not significant. Similar trends were observed for the Batch B 100 mg/mL and 50 mg/mL solutions. The osmolality and pH of the 50 mg/mL were between those of the 20 mg/mL and 100 mg/mL solutions.

The retention time of the HCQS peak in the HPLC chromatogram was about 8 min. Table 2 shows the regression equations of the calibration curves with the mean slopes and y-intercept. They were obtained using fresh standard solutions over 11 and 14 days with deionised water and 50:50 *v*/*v* methanol/water as the diluent, respectively. The standard curves were similar between the days and were linear (r^2^ ≈ 1) from 6.25 to 1000 µg/mL. The detection and quantitation limits were derived by Equations (3) and (4), respectively [55]. The values of slope featured in these equations were taken to be the mean slopes shown in Table 2.

Detection limit = (3.3 × Standard deviation of the y-intercepts)/Slope(3)

Quantitation limit = (10 × Standard deviation of the y-intercepts)/Slope(4)

The HPLC method was more sensitive with deionised water as the diluent, as shown by the lower detection and quantitation limits (Table 2). This is interesting to note because the USP recommends 50:50 *v*/*v* methanol/water as the diluent.

### 3.2. Recovery of HCQS from SureGard Filters

The recovery of HCQS from the spiked SureGard filters using deionised water and 50:50 *v*/*v* methanol/water is presented in Table 3. Deionised water was more efficient than the co-solvent for extracting HCQS from the filters. It obviated the need for the 30-min standing time to allow the filter to soak before shaking. Drug adsorption to the filter was appreciable at the low spiked drug level, as 4–5% of drug could not be recovered even when water was used. This was even more significant (11–12%) with the co-solvent. Therefore, deionised water was better for assaying the drug from SureGard filters.

### 3.3. Dose Output

The nebulisation duration of the 1 mL loaded dose runs is shown in Table 4. The correlation between solute concentration and nebulisation duration was non-linear. The nebulisation times with 1.5 mL of 100 mg/mL were understandably longer than with 1 mL.

**Figure 2:**
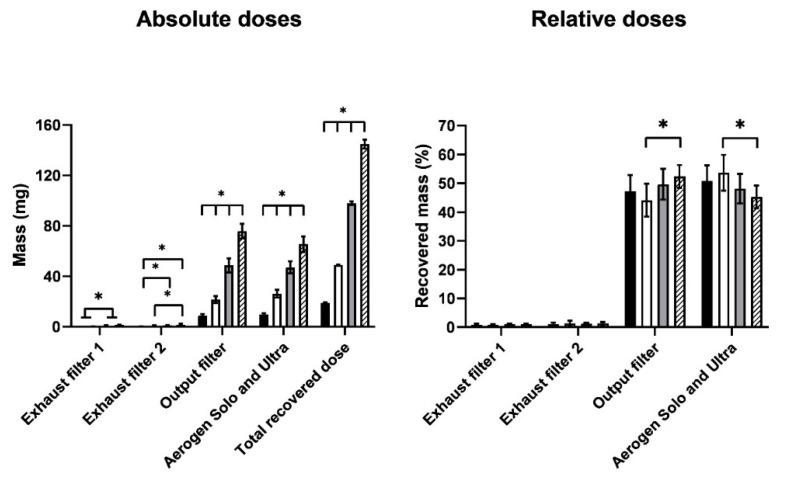
Absolute and relative doses of HCQS on the various parts of the dose output setup. The four bars represent 1 mL of 20 mg/mL (black), 1 mL of 50 mg/mL (white), 1 mL of 100 mg/mL (gray), and 1.5 mL of 100 mg/mL (hatch). Data presented as mean ± standard deviation (*n* = 9). Statistical difference indicated by * (*p* < 0.05).

The recovered dose for the runs was generally 95–105% of the loaded dose, so drug recovery was satisfactory. The absolute and relative doses with respect to the recovered dose for the various parts of the experimental setup are shown in Figure 2. The absolute dose was the assayed HCQS dose expressed in milligrams, whereas the relative dose was the assayed HCQS dose expressed as a percentage of the recovered dose. The data for the absolute doses showed that the amount of drug reaching the exhaust filters was very low. Most of the drug was shared between the output filter and the Aerogen Solo/Ultra. The output dose was approximately proportional to the loaded dose. This was confirmed by the similar distributions of relative doses on the various parts of the experimental setup between the concentration/volume combinations. The amount of drug that exited the nebuliser setup during the exhalation phase of the breathing cycle (i.e., those collected on the two exhaust filters) was low, with <2% of the recovered dose on each filter. About half of the recovered dose was emitted onto the output filter, which represented the amount of HCQS that a patient would inhale, assuming that the simulated breathing cycle is representative of the patient’s breathing; the remainder was retained in the Aerogen Solo/Ultra. The output with respect to the recovered dose from 1 mL of 50 mg/mL was slightly lower than that from 1.5 mL of 100 mg/mL (Figure 2). This is explained by the correspondingly higher drug retention in the Aerogen Solo/Ultra.

### 3.4. Laser Diffraction

The droplet size distributions measured by laser diffraction were stable over the entire measurement period for each run. The nebulisation duration (Table 5) was longer than the actual measurement time because the aerosol concentrations were low (“thin” aerosols) at the start and end of nebulisation, so the laser signal transmission at these times was higher than the trigger threshold for measurement (99.9%). The measurements generally started a few seconds after aerosols appeared in the measurement zone for all drug concentrations/volumes. The thin aerosol tailing near the end of nebulisation (i.e., thin aerosols in the measurement zone but no sizing was triggered) took about 30 s and was particularly longer (up to 1 min) for 20 mg/mL.

**Figure 3:**
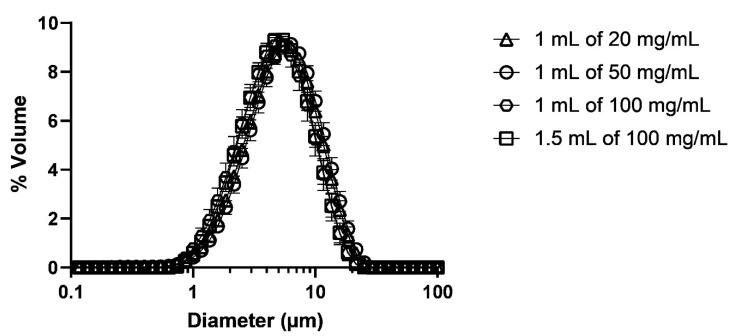
Droplet size distributions measured by laser diffraction. Data presented as mean ± standard deviation (*n* = 9).

**Figure 4:**
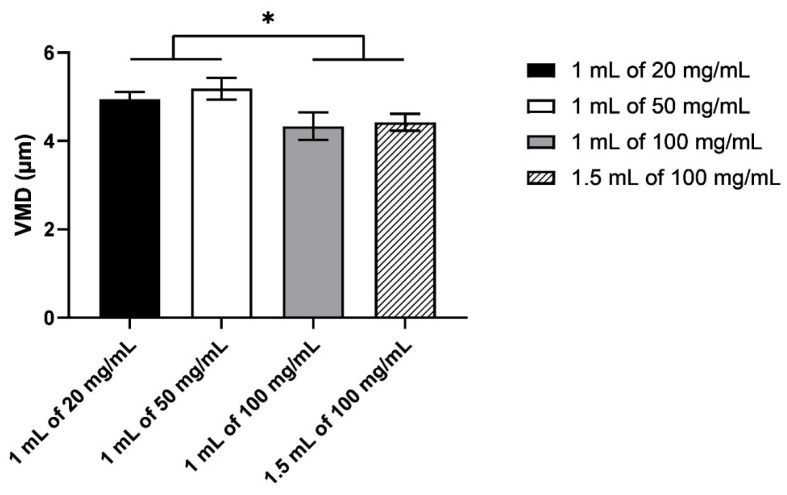
The volumetric median diameter of the droplets measured by laser diffraction. Data presented as mean ± standard deviation (*n* = 9). Statistical difference indicated by * (*p* < 0.05).

**Figure 5:**
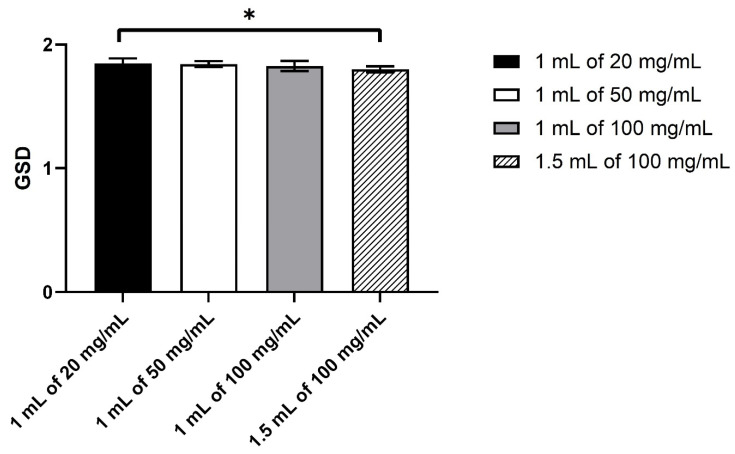
The geometric standard deviation of the droplets measured by laser diffraction. Data presented as mean ± standard deviation (*n* = 9). Statistical difference indicated by * (*p* < 0.05).

The size distributions were all monomodal and reproducible between the three nebulisers, with the peak at about 5 µm (Figure 3). There was a slight shift in the distribution to the smaller size between 100 mg/mL (both volumes) and the other two HCQS concentrations. This difference was more obvious in the VMD (Figure 4). Although the VMD for all concentrations/volumes was between 4.3 and 5.2 µm, the droplets produced from 100 mg/mL solutions were slightly but significantly (*p* < 0.05) smaller than those from 20 and 50 mg/mL (Figure 4). The GSD was relatively consistent between the four concentrations/volumes, at about 1.8 (Figure 5). However, the GSD from 1.5 mL of 100 mg/mL was also slightly but significantly (*p* < 0.05) lower than that from 1 mL of 20 mg/mL.

**Figure 6:**
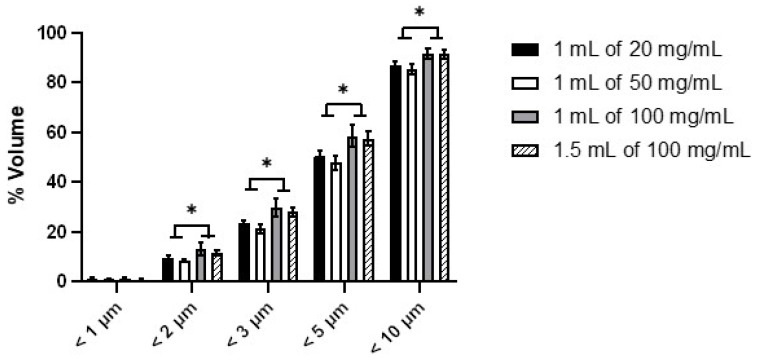
The percentage of aerosol by volume under 1, 2, 3, 5, and 10 µm measured by laser diffraction. Data presented as mean ± standard deviation (*n* = 9). Statistical difference indicated by * (*p* < 0.05).

The percentage of aerosol sample by volume under 1, 2, 3, 5, and 10 µm is shown in Figure 6. About 50–60% of the aerosols was <5 µm. The nebulisers produced minimal submicron droplets at all concentrations/volumes, but the 100 mg/mL solution consistently produced more droplets by volume than 20 and 50 mg/mL at all cutoff diameters. In other words, the droplets from the 100 mg/mL solution were smaller than those from the other two solutions. No clear dependence between droplet size and relative humidity was observed, so the difference in droplet size was attributed to the solute concentration and the resultant changes in the physicochemical characteristics of the solutions.

### 3.5. Cascade Impaction

**Figure 7:**
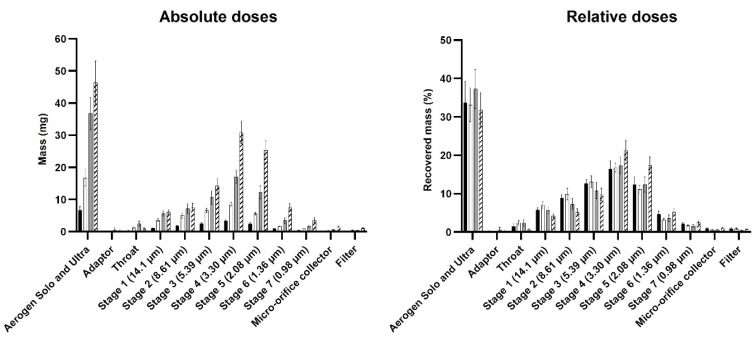
Absolute and relative doses of HCQS on the various parts of the NGI setup. The four bars represent 1 mL of 20 mg/mL (black), 1 mL of 50 mg/mL (white), 1 mL of 100 mg/mL (gray), and 1.5 mL of 100 mg/mL (hatch). Data presented as mean ± standard deviation (*n* = 3).

The nebulisation durations of the NGI runs (Table 6) were similar to those for the dose output runs (Table 4). The recovered dose was close to the loaded dose for all the runs, so drug recovery was satisfactory. The absolute and relative doses (with respect to the recovered dose) for the various parts of the setup are shown in Figure 7. Only a small amount of HCQS (<1%) was collected on the post-NGI filter, so the NGI captured practically all the emitted doses. About 30–40% of the recovered dose remained in the nebulisers after the NGI runs, compared to 50% after the dose output runs (Figure 7 and Figure 2, respectively). This might be due to the vacuum pump continuously removing droplets from the Aerogen Ultra into the NGI rather than blowing them back repeatedly into the aerosol chamber, as in the case of the breath simulator during the exhalation phase employed in the delivered dose experiments. The overall aerosol performance profiles were similar between the concentrations/volumes, with minimal throat deposition (Figure 7). However, 1.5 mL of 100 mg/mL showed more drug on Stages 4 and 5, so there was a higher proportion of fine droplets.

The emitted dose, FPD, FPF < 5 µm loaded, FPF < 5 µm emitted, MMAD emitted, and GSD emitted derived from the NGI data are summarised in Table 7, together with other key parameters measured in the dose output and laser diffraction experiments for comparison. The overall dose output rate was calculated by dividing the dose of HCQS collected in the output filter by the duration of nebulisation in the dose output experiments. The higher FPF and MMAD of 1.5 mL of 100 mg/mL also indicate that it produced smaller droplets than the other concentrations/volumes. The GSD gradually decreased with increasing concentration/volume, so the size distribution became narrower. These trends were also observed in the VMD and GSD in the laser diffraction data (Table 7). The doses collected in the output filter in the dose output experiments were consistently lower than the emitted doses in cascade impaction. This was because the vacuum pump in the latter constantly pulled the aerosol out of the nebuliser (continuous “inhalation”), and the breath simulator in the former generated a sinusoidal flow (periodic “inhalation” and “exhalation”).

### 3.6. Comparison of Dose Output, Laser Diffraction, and Cascade Impaction Data

Laser diffraction and cascade impaction data were compared to check their correlation because cascade impaction measurements can be affected by droplet evaporation in the entrained dilution air [56,57]. To improve the accuracy of the comparison, the major parameters from laser diffraction (VMD and %V < 1, 2, 3, 5, 10 µm) were converted to their volumetric aerodynamic diameters by Equation (2). The density of the 20, 50, and 100 mg/mL HCQS solutions were measured to be 1.008, 1.019, and 1.033 g/cm^3^, respectively, and were used in the calculation. The data are shown in Table 8. Correlation between the two techniques was reflected in the percent ratio of each parameter, which was the quotient of a given parameter measured by cascade impaction and that by laser diffraction. The FPFs measured by cascade impaction for all concentrations/volumes were consistently higher than those by laser diffraction at the corresponding cutoff diameters (Table 8). By the same token, the MMAD with respect to the emitted dose measured by cascade impaction was smaller than the volumetric median aerodynamic diameter (VMAD) by laser diffraction. The smaller particle sizes measured by cascade impaction could be attributed to droplet evaporation in the NGI. Despite this, its width remained relatively constant. The deviation between the corresponding GSDs was 97–110%, indicating that evaporation in the NGI was a monotonous shift to the smaller sizes without changing the width of the distribution. The main trend observed in the FPFs and %V undersize was that the lower the cutoff diameter, the larger the deviation between the two datasets, with relatively close agreement at 10 µm (97–104% deviation), to > 120% deviation at 5 µm, and > 200% deviation at 2 µm. This was most likely due to the faster evaporation rates of small droplets, which increased the FPF to a greater extent at the lower cutoff sizes. In addition, the deviation between the two datasets decreased with increasing HCQS concentration for the 1 mL solutions. This might be due to the reduction in vapour pressure with increasing HCQS concentration, which decreased the evaporation rate.

## 4. Discussion

The ClinicalTrials.gov (accessed on 20 May 2021) database records several clinical studies on inhaled hydroxychloroquine for COVID-19 at various stages of progress, from recently completed to not yet recruiting [58]. There are Phase 1 safety, tolerability, and pharmacokinetic studies on healthy subjects (ClinicalTrials.gov Identifier: NCT04461353, NCT04497519, NCT04697654), as well as efficacy studies on COVID-19 patients (NCT04477083, NCT04731051), but no results from the completed trials were available as of 17 July 2021. The formulations investigated included nebulised solutions (NCT04461353, NCT04731051), nebulised liposomal suspension (NCT04697654), and inhaled dry powders (NCT04497519, NCT04477083).

Inhaled hydroxychloroquine solutions had been tested on humans in the past. HCQS (50 µL of 100 mg/mL) aerosolised from the AERx^®^ Pulmonary Delivery System was studied more than a decade ago in Phase 1 and Phase 2 clinical trials for asthma treatment, owing to its anti-inflammatory properties [40]. Subjects inhaling nebulised 20 mg or 50 mg HCQS experienced only mild adverse effects (altered sense of taste and dizziness), with minimal influence on pulmonary and cardiac functions [48]. Two researchers recently self-tested the effects of inhaled hydroxychloroquine by inhaling a nebulised solution (1 mg in 2 mL of normal saline) twice a day [25]. The dose was increased gradually to 4 mg daily over one week. It was also well-tolerated, with the only notable adverse effect being a bitter aftertaste that remained in the mouth for up to 3 h after inhalation [25]. HCQS is bitter [59], which can affect the palatability of the nebulised solutions as the volume delivered is much larger than that from an AERx system. The consumption of peanut butter and hazelnut chocolate spread immediately after oral administration of a bitter drug such as ritonavir has been shown to decrease the duration and intensity of the aftertaste [60]. This practice may be considered after inhaling nebulised HCQS solutions. Alternatively, taste-masking liposomes loaded with the drug may be used instead, but its formulation and drug release profile are more complex [61]. A liposomal HCQS formulation delivered to Sprague–Dawley rats by intratracheal instillation showed higher dose and longer drug retention in the lungs, as well as lower systemic exposure, compared to intravenous injection of unformulated HCQS [62].

Aerogen Solo is a vibrating mesh nebuliser that can be used directly with a mouthpiece/face mask or integrated into breathing circuits with a ventilator/nasal cannula [63]. The Aerogen Ultra acts as an aerosol holding chamber used in conjunction with the Aerogen Solo. It has a port for external air entrainment and optional supplemental oxygen, where an Exhaust filter 2 was connected (Figure 1). The versatility of the nebuliser setup is an advantage for treating COVID-19 because it can be used in patients with various degrees of breathing ability, depending on the severity of the disease. It is also better than jet and ultrasonic nebulisers because the temperature and solute concentration of the liquid in the reservoir remain constant during nebulisation [64]. Its atomisation mechanism is efficient, as the residual volume at the end of nebulisation is only about 50 µL [64]. This nebuliser had been used to administer HCQS to adult subjects in a recent Phase 1 pharmacokinetic study [48]. Therefore, Aerogen Solo was chosen for nebulising the HCQS solutions in the current study.

SARS-Cov-2 is primarily transmitted by the dispersion of bioaerosols from the patient through breathing, speaking, coughing, or sneezing [65]. There is a widely held concern that patients using nebulisers can increase the spread of respiratory pathogens through exhaling contaminated droplets into the surroundings. Nebulisers were consequently banned in Hong Kong during the SARS outbreak there in 2003 [65]. Jet nebulisers would have been the most common nebulisers used at that time. Normally, nebulisers should not produce virus-laden droplets unless they and/or the nebulised liquid are contaminated. The liquid reservoir of jet nebulisers is open to the inhalation/exhalation pathway, so it can be contaminated by bioaerosols from the patient being blown into it or by the materials from the patient’s mouth dripping into the nebuliser bowl, thereby generating infected droplets that may escape into the environment if such aerosol leaves the nebuliser system while the patient is exhaling. Furthermore, not all inhaled nebulised droplets will deposit in the lungs. Some of them may be exhaled after a brief stay in the airways. It is still controversial whether these initially virus-free nebulised droplets can become contaminated while inside the airways before they are exhaled. One view is that if an inhaled droplet deposits onto the mucosal lining of the respiratory tract, then it would coalesce and fuse with the airway surface fluid so it cannot be exhaled [65]. For droplets that entered the airways but have not deposited, they should remain virus-free when they are exhaled because they did not contact the infected airway surface [65]. However, it may also be possible that during transit, those initially clean droplets coalesce with virus-laden droplets that are naturally produced in the airways, without contacting the mucosal surface. Then, these contaminated droplets may be exhaled into the surroundings. Whatever the situation is, precaution should be taken when healthcare workers and patients use nebulisers to prevent contamination of the equipment and environment. Both the exhaled aerosol as well as the aerosol that is generated but not inhaled while the patient is exhaling is filtered out by the exhaust filter on the Aerosol Solo Ultra. Exhaust filters should be placed in the manner adopted in the dose output experiments to capture all exhaled droplets (Figure 1). Their collection efficiency was excellent because drug recovery was near 100%. Additionally, the nebulisers should be disinfected thoroughly before and after use.

Filtration of the HCQS solutions through a 0.22 µm syringe filter did not affect the drug concentration, so this could be a suitable sterilisation method. The osmolality and pH of the solutions were controlled because acidity and non-isotonicity may trigger bronchoconstriction and cough [51,66]. The osmolality of normal saline and physiological plasma are 286 and 288 mOsmol/kg H_2_O, respectively [67]. The osmolality of the normal saline used for dilution in the current study conformed to this range. The osmolality of inhaled solutions should preferably be <320 mOsmol/kg H_2_O [66]. After filtration, the osmolality of the HCQS solutions decreased to near or below this level, so they are suitable (Table 1). The airway surface liquid in conducting airways and the alveolar subphase fluid in alveoli have a pH of 6.9 [68]. However, they become more acidic when the lungs are infected and inflamed, with a pH reduction of at least 0.2 from baseline in pneumonia [69]. Airway surface fluid has substantial buffering capacity so that the deposition of unbuffered aerosols, such as the HCQS solutions, would only induce a transient pH change that can quickly be restored [66]. Since the pH of the filtered HCQS solutions was near-neutral (7.03–7.25) (Table 1), disturbance to the pH in the airways should be minimal.

Since chloroquine binds to glass and plastics [70,71], the potential adsorption of hydroxychloroquine to surfaces of the containers and experimental setup in the study was checked in preliminary experiments. The concentration of the HCQS solutions was unchanged after contacting surfaces in Aerogen Solo and Ultra (data not shown). On the other hand, adsorption was observed in the SureGard filters, especially with the lower level of spiked drug (Table 3). The ability of recovering the drug in the assay was different between deionised water and 50:50 methanol/water. This could be due to a difference in the affinity of HCQS between the filter material and the washing liquid. This is analogous to the situation in a HPLC column between the stationary and mobile phases. HCQS was more easily extracted from SureGard filters by water than the co-solvent. The difference in the extraction power of the liquids was more prominent at low drug concentrations because the liquids had to compete with drug adsorption on the filter. The recovery could be improved by soaking the filters with the liquids for 30 min before shaking (Table 3). However, the absolute amount not recovered from 2 mg was only about 0.1 mg for water and 0.24 mg for the co-solvent. These constitute <1.5% of the loaded dose in the nebulisers for the concentrations/volumes investigated in this project so the resultant error in the total recovery was insignificant. Therefore, the final results of the filters did not require correction. On the other hand, recovery from spiked NGI impactor stages with 50:50 methanol/water was high (data not shown) so drug adsorption on the metallic surfaces was negligible. Therefore, the co-solvent was used for the cascade impaction experiments because it is recommended by the USP. The rationale was to follow the USP HPLC method unless there was a need for minor adjustments. Thus, deionised water was used instead for the dose output experiments to maximise recovery because the droplets were collected by SureGard filters rather than metallic impactor stages.

The nebulisation duration in the laser diffraction experiments (Table 5) were longer than those in the dose output (Table 4) and cascade impaction experiments (Table 6), especially for 20 mg/mL. Although the determination of the end of nebulisation was different between these experiments (the aerosol cloud could be easily observed visually in laser diffraction but it could not be seen clearly in the dose output and cascade impaction experiments), it could not account for the doubling of nebulisation duration for 20 mg/mL. It might be due to an effect of lower airflow through the Aerogen Ultra in the laser diffraction experiments and a potential augmented influence from solute concentration on lengthening nebulisation duration.

The HCQS solutions at all concentrations/volumes could form inhalable droplets, but the droplet sizes measured by cascade impaction were consistently smaller than those by laser diffraction, with smaller MMADs and higher FPFs (Table 8). The USP recommends pre-chilling the NGI to minimise droplet evaporation [52]. However, despite that, evaporation could still occur because the dilution air drawn into the impactor was ambient, which was warmer and non-humidified [57,72,73,74]. The USP does not require the use of humidified air, nor are the nebulisers clinically used with humidified air. However, air is humidified rapidly as it enters the airways, so inhaled isotonic droplets should not evaporate significantly [75]. Therefore, laser diffraction data would better reflect their true size distribution because the measurement zone was close to the exit of the nebuliser mouthpiece, where there was minimal evaporation [72].

Droplet size was observed to decrease with increasing HCQS concentration, especially between 50 and 100 mg/mL (Table 7 and Table 8). This trend has been reported in previous studies on vibrating mesh nebulisers [76,77,78,79]. An increase in the concentration of ionic species increased the electrical conductivity of the liquid, which then dissipated the high charges that would otherwise be present between water and the nebuliser mesh. Consequently, fluid adhesion to the mesh was reduced, and droplets could be detached easier, resulting in the production of smaller droplets [76,77,78,79]. However, the reduction in droplet size with increasing ionic concentration is sigmoidal [79]. In other words, the droplet size will reach a plateau after the ionic concentration exceeds a threshold. The threshold concentration is dependent on the ionic species and liquid vehicle and beyond which other physicochemical factors (e.g., viscosity and surface tension) may then become dominant in affecting droplet size [76,77,78,79]. It has been reported that droplet size from vibrating mesh nebulisers decreases with viscosity [76,77,78]. It should be noted that any factors that affect droplet size may also affect the aerosol output rate. The electrical conductivity, viscosity, and surface tension of the HCQS solutions were not measured in the current study, but the 100 mg/mL solutions were qualitatively more viscous than the 20 and 50 mg/mL solutions. Thus, it is unknown which of the aforementioned physicochemical factors exerted the most effect on the droplet size in the present study.

The in vivo antiviral and anti-inflammatory concentrations of hydroxychloroquine in the airways is unknown, but its in vitro antiviral EC_50_ is approximately 1–5 µM, as discussed in the Introduction. After inhaling a nominal dose of 50 mg HCQS from an Aerogen nebuliser in a Phase 1 study, the peak respiratory tissue concentration was predicted by pharmacokinetic modelling to reach 500 µM, which was at least 100-fold higher than the in vitro antiviral EC_50_ [48]. It decreased to 10 µM at 24 h post-inhalation but was still at least double the in vitro EC_50_. This supports the feasibility of treating COVID-19 with nebulised HCQS solutions. High drug levels in the airways can be maintained by multiple daily inhalations. Idkaidek et al. employed a physiologically based pharmacokinetic model to estimate the inhaled dose needed for COVID-19 based on this concentration range [80]. The model featured droplets with a VMD of 5.6 µm. The proportions depositing in the trachea, bronchioles, and alveoli were 10, 13, and 30% by mass, respectively [80]. Their sum (53%) could be interpreted as the proportion of the emitted aerosol < 5 µm because they theoretically deposited in the lungs. It was found that inhaling 25 mg of hydroxychloroquine twice a day could achieve a maximum concentration (C_max_) ≥ 7 µM in the various parts of the lungs, while the plasma C_max_ was only 0.18 µM [80]. If the inhaled dose was doubled to 50 mg hydroxychloroquine twice a day, then the lung C_max_ reached ≥ 13 µM and plasma C_max_ increased to 0.35 µM. Thus, pulmonary drug concentrations higher than the in vitro antiviral EC_50_ with low systemic absorption are potentially achievable. The plasma hydroxychloroquine concentration for rheumatoid arthritis treatment is typically < 1 µM, while serious toxicity was associated with plasma levels from 2.05 to 18.16 µM [81]. Therefore, systemic adverse effects should be minimal with the low plasma concentrations from the inhalation regimens outlined above. The emitted dose obtained from the dose output experiments was 9.1–75.9 mg (Table 7), depending on the concentration/volume of the HCQS solution. This encompassed the range of 25–50 mg hydroxychloroquine (equivalent to 32.3–64.5 mg HCQS) proposed by Idkaidek et al. The VMD of our droplets were 4.3–5.2 µm, with 50–60% of them < 5 µm (Table 7, Figure 6), so they were slightly smaller than those used in their model. This suggests that if our HCQS aerosols were inhaled twice a day, especially with the two volumes of 100 mg/mL HCQS solutions (dose outputs of 48.8–75.9 mg), they may be able to produce pulmonary drug concentrations above the in vitro antiviral EC_50_. To put this into perspective, 400–600 mg of HCQS were delivered orally per day in previous COVID-19 clinical trials [30]. This is 5–12-fold higher than the emitted doses from the two volumes of 100 mg/mL HCQS solutions. Yao et al. proposed several oral delivery regimens involving hundreds of milligrams HCQS taken twice to four times daily using physiologically based pharmacokinetic modelling to maintain sufficiently high ratios of free lung tissue trough concentration to the in vitro EC_50_ (R_LTEC_), in the range of 21–169 [27]. The higher the R_LTEC_, the more likely in vivo antiviral activity is achieved [45]. However, it was found that the free lung trough concentration used by Yao et al. included the in vivo intracellular drug concentration, which is expected to much higher than the in vivo extracellular drug concentration because HCQS highly accumulates in acidic cellular organelles (e.g., endosomes and lysosomes) [45]. Since the reported in vitro EC_50_ was extracellular, Yao et al. overestimated the R_LTEC_. The corrected R_LTEC_ was much lower after recalculation with the in vivo extracellular drug concentration, ranging from 0.11 to 0.34 [45], which was deemed too low for in vivo antiviral efficacy. Moreover, the risk of systemic adverse effects from the high oral doses outweighed the low to lack of therapeutic effects observed in clinical studies [17,18,30]. Therefore, inhalation would be more efficient and safer for potential treatment of COVID-19.

As mentioned above, intratracheal liposomal HCQS has been tested on rats [62]. There is also a human clinical trial on a nebulised liposomal HCQS suspension (NCT04697654) [58]. Therefore, formulating the drug as simple, aqueous solutions is not the only approach. Multivalent nanoparticles functionalised with ligands targeting SARS-CoV-2 and/or receptors for cellular infection have been proposed for COVID-19 treatment, owing to their engineering flexibility and versatility [82]. They can be designed to target multiple pathogenic pathways of the disease. Multivalent nanoparticles may be employed to deliver otherwise toxic compounds such as oncology drugs (e.g., erlotinib and sunitinib), which may prevent viral entry into cells by inhibiting AP2-associated protein kinase 1 [82]. Systemic adverse effects are minimised due to the specificity offered by the functional ligands. However, although the number of approved nanoparticle products has been growing, their development takes years to decades, as they have unique technical, safety, and regulatory challenges [83]. Moreover, complex constructs such as multivalent nanoparticles may become damaged by the shear and stress during aerosolisation so they may not be a straightforward formulation option. Given the extremely urgent need to develop readily accessible and affordable therapy for the prophylaxis and treatment of COVID-19 for ambulatory and hospitalised patients, including those who may require ventilators, repurposing a well-established drug with antiviral and anti-inflammatory activities that has been used clinically, such as HCQS, is an attractive path to the rapid development of safe and effective treatments. Indeed, the United States and European Union have abbreviated regulatory pathways for drugs previously approved for human use. In addition, the risk of inhaled aqueous HCQS solutions is very low because of the reports of good safety and tolerability, even in patients with pulmonary disease (see above). Therefore, the translational and clinical barriers are significantly lower. The risk of failure due to safety problems is also much reduced.

## 5. Conclusions

Inhalable droplets of isotonic and pH-neutral HCQS solutions generated from vibrating mesh nebulisers were characterised. Droplet size decreased with increasing solute concentration. A range of emitted and fine particle doses were obtained with 20–100 mg/mL HCQS, with the 100 mg/mL solution potentially able to achieve sufficiently high drug concentrations in the airways for antiviral effects of COVID-19 with low systemic absorption.

## Figures and Tables

**Table 1:** Drug concentration, osmolality, and pH of HCQS solutions before and after filtration.

	Batch A		Batch B	
	100 mg/mL	20 mg/mL	100 mg/mL	50 mg/mL
**Before filtration**				
**Concentration (mg/mL)**	Not measured	20.3 ± 0.2	100.8 ± 0.8	50.8 ± 0.5
**Osmolality (mOsmol/kg H_2_O)**	323.0 ± 6.6	286.5 ± 6.6	323.5 ± 14.2	315.5 ± 3.4
**pH**	7.19	7.38	7.09	7.15
**After filtration**				
**Concentration (mg/mL)**	Not measured	20.0 ± 0.3	99.1 ± 1.1	49.9 ± 0.9
**Osmolality (mOsmol/kg H_2_O)**	314.0 ± 4.7	275.3 ± 7.2	321.3 ± 13.4	299.3 ± 5.6
**pH**	7.16	7.25	7.03	7.11

Osmolality is presented as mean ± standard deviation (*n* = 4). One pH measurement was made for each solution (*n* = 1).

**Table 2:** HPLC calibration curves, detection limit, and quantitation limit for HCQS from 6.25 to 1000 µg/mL.

	Regression Equation	Detection Limit (µg/mL)	Quantitation Limit (µg/mL)
**Deionised water**	A = 43,294C + 32,746	2.7	8.3
**50:50 *v/v* methanol:water**	A = 42,848C − 19,037	7.2	21.7

A = Peak area at 254 nm. C = HCQS concentration in µg/mL. The slopes and y-intercepts were the mean of 11 and 14 values for water and the co-solvent, respectively.

**Table 3:** Recovery of HCQS from spiked SureGard filters.

		Deionised Water	50:50 *v/v* Methanol/Water
**2 mg HCQS**	**Immediate 5 min shaking**	95.7%	87.3%
	**30 min standing, 5 min shaking**	95.3%	88.9%
**75 mg HCQS**	**Immediate 5 min shaking**	100.7%	94.4%
	**30 min standing, 5 min shaking**	100.2%	99.3%

One measurement was performed for each scenario (*n* = 1).

**Table 4:** Nebulisation duration of the dose output experiments.

	Nebuliser 1	Nebuliser 2	Nebuliser 3	All Nebulisers
**1 mL, 20 mg/mL**	3 min 26 s ± 7 s	4 min 27 s ± 31 s	3 min 41 s ± 18 s	3 min 51 s ± 33 s
**1 mL, 50 mg/mL**	5 min 4 s ± 13 s	5 min 7 s ± 10 s	5 min 8 s ± 16 s	5 min 6 s ± 11 s
**1 mL, 100 mg/mL**	6 min 24 s ± 26 s	6 min 25 s ± 35 s	6 min 22 s ± 6 s	6 min 24 s ± 22 s
**1.5 mL, 100 mg/mL**	10 min 55 s ± 43 s	9 min 31 s ± 36 s	10 min 24 s ± 45 s	10 min 17 s ± 52 s

Data presented as mean ± standard deviation (*n* = 3 for Nebulisers 1, 2, and 3; *n* = 9 for all nebulisers).

**Table 5:** Nebulisation duration of the laser diffraction experiments.

	Nebuliser 1	Nebuliser 2	Nebuliser 3	All Nebulisers
**1 mL, 20 mg/mL**	6 min 1 s ± 25 s	5 min 54 s ± 17 s	6 min 7 s ± 15 s	6 min 1 s ± 18 s
**1 mL, 50 mg/mL**	6 min 24 s ± 22 s	6 min 50 s ± 8 s	6 min 32 s ± 3 s	6 min 35 s ± 16 s
**1 mL, 100 mg/mL**	7 min 4 s ± 3 s	6 min 51 s ± 13 s	6 min 55 s ± 3 s	6 min 56 s ± 9 s
**1.5 mL, 100 mg/mL**	11 min 42 s ± 36 s	11 min 23 s ± 31 s	11 min 19 s ± 45 s	11 min 28 s ± 34 s

Data presented as mean ± standard deviation (*n* = 3 for Nebulisers 1, 2, and 3; *n* = 9 for all nebulisers).

**Table 6:** Nebulisation duration of the cascade impaction experiments.

	Nebuliser 1	Nebuliser 2	Nebuliser 3	All Nebulisers
**1 mL, 20 mg/mL**	3 min 48 s ± 7 s	3 min 50 s ± 44 s	3 min 32 s ± 22 s	3 min 44 s ± 26 s
**1 mL, 50 mg/mL**	5 min 43 s ± 9 s	5 min 15 s ± 19 s	5 min 35 s ± 10 s	5 min 31 s ± 17 s
**1 mL, 100 mg/mL**	6 min 41 s ± 42 s	6 min 26 s ± 33 s	6 min 18 s ± 21 s	6 min 28 s ± 30 s
**1.5 mL, 100 mg/mL**	10 min 48 s ± 20 s	9 min 53 s ± 48 s	9 min 6 s ± 26 s	9 min 56 s ± 53 s

Data presented as mean ± standard deviation (*n* = 3 for Nebulisers 1, 2, and 3; *n* = 9 for all nebulisers).

**Table 7:** Summary of the key parameters measured in the dose output, laser diffraction, and cascade impaction experiments.

	1 mL of 20 mg/mL	1 mL of 50 mg/mL	1 mL of 100 mg/mL	1.5 mL of 100 mg/mL
**Dose output**				
**Dose collected in output filter (mg)**	9.05 ± 0.96	21.67 ± 2.81	48.76 ± 5.57	75.88 ± 5.87
**Overall dose output rate (mg/min)**	2.38 ± 0.38	4.25 ± 0.57	7.63 ± 0.82	7.42 ± 0.71
**Laser diffraction**				
**VMD (µm)**	4.95 ± 0.17	5.19 ± 0.25	4.34 ± 0.31	4.42 ± 0.19
**GSD**	1.85 ± 0.04	1.84 ± 0.02	1.83 ± 0.04	1.80 ± 0.02
**Cascade impaction**				
**Emitted dose (mg)**	13.2 ± 1.15	33.8 ± 2.19	61.9 ± 5.28	99.0 ± 5.71
**FPD < 5 µm (mg)**	9.49 ± 1.13	22.7 ± 1.34	44.2 ± 5.60	81.6 ± 6.93
**FPF < 5 µm loaded (%)**	47.3 ± 5.64	45.3 ± 2.74	44.2 ± 5.60	54.4 ± 4.57
**FPF < 5 µm emitted (%)**	71.8 ± 3.44	67.3 ± 3.25	71.3 ± 4.71	82.3 ± 3.02
**MMAD emitted (µm)**	3.00 ± 0.18	3.27 ± 0.25	2.99 ± 0.27	2.50 ± 0.17
**GSD emitted**	2.02 ± 0.06	1.94 ± 0.14	1.88 ± 0.05	1.75 ± 0.05

Data presented as mean ± standard deviation (*n* = 9).

**Table 8:** Comparison of cascade impaction and laser diffraction data.

1 mL of 20 mg/mL				
Cascade Impaction Data		Laser Diffraction Data (Aerodynamic Diameter)		Cascade Impaction Data/Laser Diffraction Data (%)
**FPF emitted < 1 µm (%)**	6.28 ± 0.53	**%V < 1 µm**	1.17 ± 0.53	638.81 ± 277.78
**FPF emitted < 2 µm (%)**	29.49 ± 0.95	**%V < 2 µm**	9.79 ± 0.95	305.00 ± 51.55
**FPF emitted < 3 µm (%)**	50.18 ± 1.29	**%V < 3 µm**	23.54 ± 1.29	214.19 ± 24.19
**FPF emitted < 5 µm (%)**	71.76 ± 1.99	**%V < 5 µm**	50.69 ± 1.99	141.88 ± 10.90
**FPF emitted < 10 µm (%)**	90.88 ± 1.78	**%V < 10 µm**	86.94 ± 1.78	104.60 ± 3.58
**MMAD emitted (µm)**	3.00 ± 0.17	**VMAD (µm)**	4.97 ± 0.17	60.45 ± 5.11
**GSD emitted**	2.02 ± 0.04	**GSD**	1.85 ± 0.04	109.53 ± 4.91
**1 mL of 50 mg/mL**				
**FPF emitted < 1 µm (%)**	4.96 ±0.30	**%V < 1 µm**	1.05 ± 0.30	506.31 ± 166.86
**FPF emitted < 2 µm (%)**	24.48 ± 0.67	**%V < 2 µm**	8.63 ± 0.67	286.29 ± 46.16
**FPF emitted < 3 µm (%)**	45.29 ± 1.73	**%V < 3 µm**	21.46 ± 1.73	212.77 ± 28.92
**FPF emitted < 5 µm (%)**	67.30 ± 2.83	**%V < 5 µm**	47.93 ± 2.83	140.96 ± 12.42
**FPF emitted < 10 µm (%)**	88.34 ± 2.04	**%V < 10 µm**	84.86 ± 2.04	104.17 ± 3.42
**MMAD emitted (µm)**	3.27 ± 0.25	**VMAD (µm)**	5.23 ± 0.25	62.77 ± 6.63
**GSD emitted**	1.94 ± 0.02	**GSD**	1.84 ± 0.02	105.51 ± 7.74
**1 mL of 100 mg/mL**				
**FPF emitted < 1 µm (%)**	4.68 ± 0.36	**%V < 1 µm**	1.31 ± 0.36	373.53 ± 95.63
**FPF emitted < 2 µm (%)**	27.62 ± 2.44	**%V < 2 µm**	13.28 ± 2.44	214.13 ± 50.89
**FPF emitted < 3 µm (%)**	50.65 ± 3.80	**%V < 3 µm**	29.73 ± 3.80	172.71 ± 28.55
**FPF emitted < 5 µm (%)**	71.34 ± 4.29	**%V < 5 µm**	57.62 ± 4.29	124.33 ± 11.29
**FPF emitted < 10 µm (%)**	88.44 ± 2.08	**%V < 10 µm**	91.18 ± 2.08	97.03 ± 2.54
**MMAD emitted (µm)**	2.99 ± 0.32	**VMAD (µm)**	4.39 ± 0.32	68.46 ± 7.29
**GSD emitted**	1.88 ± 0.04	**GSD**	1.83 ± 0.04	103.18 ± 4.36
**1.5 mL of 100 mg/mL**				
**FPF emitted < 1 µm (%)**	6.58 ± 0.20	**%V < 1 µm**	0.90 ± 0.20	777.58 ± 273.88
**FPF emitted < 2 µm (%)**	36.57 ± 1.04	**%V < 2 µm**	11.87 ± 1.04	309.75 ± 39.07
**FPF emitted < 3 µm (%)**	62.87 ± 1.91	**%V < 3 µm**	28.15 ± 1.91	223.94 ± 19.12
**FPF emitted < 5 µm (%)**	82.28 ± 2.74	**%V < 5 µm**	56.32 ± 2.74	146.31 ± 7.45
**FPF emitted < 10 µm (%)**	94.29 ± 1.85	**%V < 10 µm**	90.76 ± 1.85	103.92 ± 2.10
**MMAD emitted (µm)**	2.50 ± 0.19	**VMAD (µm)**	4.50 ± 0.19	55.66 ± 3.90
**GSD emitted**	1.75 ± 0.02	**GSD**	1.80 ± 0.02	97.43 ± 2.57

Data presented as mean ± standard deviation (*n* = 9).

## Data Availability

Not applicable.
